# Supplementary material for: Empirical methods for controlling false positives and estimating confidence in ChIP-Seq peaks
Source: BMC Bioinformatics. 2008 Dec 5;9:523. doi: 10.1186/1471-2105-9-523 (PMC2628906; doi:10.1186/1471-2105-9-523)
Supplement: Additional File 1 — A variety of html documents from the USeq web site detailing the available applications, their best usage, output file type descriptions, command line menus, etc. [file 1471-2105-9-523-S1.zip › USeqUserGuides/JavaDoc.zip!/JavaDoc/trans/graphics/package-use.html]

Uses of Package trans.graphics


|  |  |  |
| --- | --- | --- |
| **Overview** \| **Package** \| Class \| **Use** \| **Tree** \| **Deprecated** \| **Index** \| **Help** | |  |
| PREV   NEXT | **FRAMES**    **NO FRAMES**     **All Classes** |  |


---


## **Uses of Package trans.graphics**

| Packages that use trans.graphics | |
| --- | --- |
| **trans.graphics** | For graphically representing '.cel' files and intervals. |

Classes in trans.graphics used by trans.graphics

****CelMasker****
  
          Application to draw and mask a raw text cel file.

****GenomicRegion****

****GenomicRegionGlyph****

****Graph****
  
          Graph for `IntervalPlotter`.

****IntervalDrawPanel****
  
          Panel with the meat for `IntervalPlotter`

****RankedSetAnalysis****
  
          Performs set analysis on genomic regions with a visual display.

****VirtualCelFrame****
  
          Frame for `VirtualCel`.

---


|  |  |  |
| --- | --- | --- |
| **Overview** \| **Package** \| Class \| **Use** \| **Tree** \| **Deprecated** \| **Index** \| **Help** | |  |
| PREV   NEXT | **FRAMES**    **NO FRAMES**     **All Classes** |  |


---
